# The economic value of plasma *p* ‐tau217 testing for patients presenting with cognitive impairment in the United States

**DOI:** 10.1002/alz70861_108060

**Published:** 2025-12-23

**Authors:** Drew Spargo, Pablo Anaya, Pallavi Krishnamurthy, Christophe Sapin, James Hendrix, Samantha C. Burnham, Rose C. Beck

**Affiliations:** ^1^ Eli Lilly and Company, Indianapolis, IN USA; ^2^ IQVIA, Falls Church, VA USA

## Abstract

**Background:**

Plasma *p* ‐tau217 testing offers an accessible and minimally‐invasive way to identify amyloid pathology as part of diagnostic criteria for Alzheimer’s disease (AD).^1,2^ An ongoing clinical utility study (J4Y‐MC‐B002) is assessing the impact of *p* ‐tau217 results on physicians’ intended management and confidence in working diagnosis for patients presenting with cognitive impairment (CI). Based on J4Y‐MC‐B002 data, the current study estimated the economic value of *p* ‐tau217 testing for patients evaluated for CI from the US payer perspective.

**Method:**

A decision‐tree was constructed to represent the AD diagnostic pathway for patients (mean [standard deviation] age, 73.1 [7.2] years; 59.3% female) presenting for initial CI evaluation. A Markov model, simulating lifetime progression and treatment across AD or non‐AD CI health states (mild cognitive impairment; mild, moderate, and severe dementia) was linked to the AD diagnostic pathway. Use of plasma *p* ‐tau217 plus standard clinical evaluation (SCE) was compared to SCE alone. Baseline patient characteristics and diagnostic inputs were based on J4Y‐MC‐B002 (study scenario); a second analysis sourced prevalence of amyloid pathology and SCE accuracy from the literature (literature scenario). Outcomes included costs (diagnostic, treatment‐related, medical and non‐medical), quality‐adjusted life‐years (QALYs), incremental cost‐effectiveness ratios (ICERs), and economically justifiable prices (EJPs) at commonly‐accepted willingness to pay (WTP) thresholds.

**Result:**

In the study scenario, adding plasma *p* ‐tau217 testing to SCE reduced diagnostic costs by $342/patient, through more efficient use of amyloid PET and CSF testing. Higher lifetime costs ($274,876 vs $273,999 and QALYs (2.05 vs 2.00) among those who received vs did not receive testing were driven by more patients receiving a biomarker‐confirmed AD diagnosis and appropriate amyloid‐targeting therapy. The ICER was $17,571/QALY gained. Results were similar in the literature scenario. In the study and literature scenarios, respectively, the EJPs of plasma *p* ‐tau217 testing were $4,311 and $3,730 at a WTP of $100,000/QALY gained.

**Conclusion:**

Adding plasma *p* ‐tau217 testing to SCE for patients presenting with CI may reduce costs associated with amyloid PET imaging and AD CSF testing, offering a cost‐effective way to support AD diagnosis.

**References**

1. Arslan B, et al. *Clin Chem Lab Med*. 2024;621063‐1069.

2. Mielke MM, et al. *Alzheimers Dement*. 2024;20:8216‐8224.

